# Testing a Preference Tool in Different Care Settings in Germany: Descriptive Results

**DOI:** 10.1093/geroni/igab046.1015

**Published:** 2021-12-17

**Authors:** Johannes Bergmann, Tobias Stacke, Mike Rommerskirch-Manietta, Daniel Purwins, Christina Manietta, Armin Stroebel, Martina Roes

**Affiliations:** 1 DZNE, Witten, Nordrhein-Westfalen, Germany; 2 DZNE, German Center for Neurodegenerative Diseases, Nordrhein-Westfalen, Germany; 3 Center for Clinical Studies, Erlangen, Bayern, Germany; 4 German Center for Neurodegenerative Diseases (DZNE), Witten, Nordrhein-Westfalen, Germany

## Abstract

Background: The tool “Preferences for Everyday Living Inventory” (PELI) for Nursing Homes (NH) was developed in the USA. In our project PELI-D, the PELI was translated from English into German and piloted in three care settings: Nursing Home (NH), Home Care (HC) and Adult Day Care (AD). Objective: The objective is to provide insights in preferences of importance of older adults in need of care in Germany. Methods: Data collection was carried out in 2019 on multiple measurement points: n=48 baseline (T0) and n=41 two-week follow-up (T1). Results: The results indicate that the importance of certain preferences distinguishes between the care settings: In NH preferences for body care and aspects of professional care are important. Additionally, in HC the aspects of social contact and eating/drinking are perceived as important. Comparing T0 and T1, importance of the item’s daily routines, social contact and aspects of privacy seem to be reliable.

